# Predictive modeling of corporate financial risk and its implications for health economics and public health efficiency

**DOI:** 10.3389/fpubh.2026.1751753

**Published:** 2026-05-28

**Authors:** Wenyuan Zhi, Yao Kang

**Affiliations:** 1 Shanxi Vocational University of Engineering Science and Technology, Jinzhong, Shanxi, China; 2 Nanjing Pukou District Traditional Chinese Medicine Hospital, Nanjing, Jiangsu, China

**Keywords:** corporate financial risk, decision support systems, health economics, predictive modeling, public health efficiency, risk transmission mechanisms

## Abstract

**Introduction:**

This study investigates the relationship between corporate financial risk and public health efficiency by developing a unified analytical framework that integrates financial risk prediction with health economic outcomes. We first establish a conceptual mechanism through which corporate financial instability affects public health systems via three primary pathways: direct effects through reduced healthcare funding and resource constraints, indirect effects through employment instability and changes in insurance coverage, and systemic effects through broader macroeconomic disruptions.

**Methods:**

Building on this framework, we propose the Risk Economic Health Nexus Model (REHNM), which combines probabilistic modeling, latent variable inference, and multimodal data integration to capture the complex and uncertain relationships between financial indicators and health outcomes. To account for temporal dynamics and external influences, we further introduce the Dynamic Risk Assessment Model (DRAM), enabling the model to track evolving financial conditions and their downstream health impacts. The Strategic Risk Health Integration Approach (SRHIA) is developed to translate predictive outputs into actionable insights for policymakers and health economists.

**Results and discussion:**

Empirical results demonstrate that the proposed framework improves predictive performance while providing interpretable insights into the linkage between financial risk and public health efficiency. Importantly, the model is designed as a decision support tool that can assist in early risk detection, resource allocation, and health system planning, thereby contributing to improved economic resilience and public health outcomes.

## 1 Introduction

The predictive modeling of corporate financial risk is a critical area of research with significant implications for health economics and public health efficiency. Understanding and forecasting financial risks within corporations not only aids in safeguarding economic stability but also plays a crucial role in ensuring the efficient allocation of resources in public health sectors. Financial instability can lead to reduced funding for health initiatives, impacting the quality and accessibility of healthcare services (1). Moreover, the ripple effects of corporate financial distress can extend to broader economic challenges, affecting employment rates and public health funding. Therefore, developing robust predictive models for corporate financial risk is essential for maintaining economic health and optimizing public health strategies (2). By accurately predicting financial risks, policymakers and health economists can better allocate resources, plan for contingencies, and implement preventive measures to mitigate adverse effects on public health systems. This research area not only addresses the immediate needs of financial risk management but also contributes to the long term sustainability and efficiency of public health services (3).

To clarify the conceptual pathway examined in this study, we develop a structured framework describing how corporate financial risk may propagate to health economics and public health efficiency. We distinguish three main transmission channels. The *direct channel* operates through financial and budgetary constraints, where corporate distress reduces investment capacity, disrupts procurement, and affects the continuity of healthcare related services, thereby increasing costs and lowering operational efficiency. The *indirect channel* captures broader socioeconomic spillovers, including employment instability, income shocks, and disruptions in insurance coverage, which influence healthcare access and utilization. A *systemic channel* reflects the accumulation of firm level shocks into macroeconomic and institutional pressures, affecting public budgets, resource allocation, and health system performance (4).

From a theoretical perspective, the linkage between firm level financial risk and population level health outcomes is mediated by meso level institutional and sectoral structures (5). Financially vulnerable firms that play critical roles as healthcare providers, insurers, pharmaceutical suppliers, or major employers can transmit shocks through service delivery systems, labor markets, and fiscal networks. Rather than assuming a direct one to one mapping from firm distress to aggregate health outcomes, we conceptualize population level effects as the aggregated consequences of these intermediate mechanisms operating across regions and sectors (6). From a theoretical standpoint, this study adopts a layered transmission view in which firm level financial risk is treated as a microeconomic shock, while population level health outcomes are understood as emergent system level consequences of repeated and aggregated shocks transmitted through meso level institutions. This perspective is consistent with the idea that health system performance is shaped not only by aggregate fiscal conditions, but also by the stability of organizational actors embedded in service delivery, employment, insurance, and supply networks. Under this view, financially vulnerable firms matter for population health not because each distressed firm produces an immediate population wide effect, but because instability among strategically connected actors can accumulate into broader expenditure pressure, access constraints, service discontinuity, and efficiency loss across regions and sectors. From a theoretical standpoint, the present study adopts a layered transmission perspective. Corporate financial risk is treated as a micro level economic shock, while public health efficiency and health economic outcomes are understood as system level consequences that emerge only when such shocks are transmitted through meso level institutional mechanisms. These mechanisms include disruptions in healthcare related financing, employment and income instability, insurance related constraints, supply chain fragility, and fiscal pressure on service provision. Under this view, the relevance of firm level instability to public health does not arise from a direct one step causal effect, but from the cumulative and regionally distributed consequences of financially stressed actors embedded in service delivery and socioeconomic systems.

Consistent with this framework, the empirical focus of this study is on proximal system level outcomes that are more directly observable and measurable, including healthcare expenditure pressure, service accessibility, continuity of provision, and efficiency of resource utilization. More distal outcomes, such as long term population health status or inequality dynamics, are treated as downstream implications and are interpreted with caution. This conceptualization provides the foundation for the modeling strategy developed in the subsequent sections. Accordingly, the framework developed in this study encompasses both direct and indirect transmission channels, while also allowing for broader systemic spillovers. The direct dimension primarily concerns funding and operational constraints that may affect healthcare related expenditure and service continuity, whereas the indirect dimension concerns labor market, income, and insurance mediated pathways. Empirically, however, our analysis focuses on proximal system level consequences that are more readily observable in integrated data, rather than attempting to isolate the independent causal contribution of each transmission channel.

Early approaches to predictive modeling in corporate financial risk assessment primarily relied on structured frameworks that utilized predefined rules and expert driven methodologies. These systems were designed to simulate logical reasoning processes by incorporating domain specific insights into the analysis of financial data. While these methods provided a degree of interpretability and consistency, they were often constrained by their inability to adapt to the dynamic and multifaceted nature of financial markets (7). The reliance on static rules and manual input limited their scalability and effectiveness in addressing the complexities of evolving corporate risk factors. Despite these challenges, these foundational approaches underscored the importance of structured reasoning in financial risk modeling and paved the way for subsequent advancements.

To overcome the limitations of rigid frameworks, researchers began exploring algorithmic techniques capable of learning patterns directly from data. These methods introduced statistical models that could identify relationships and dependencies within financial datasets, offering greater adaptability and precision in risk prediction. Techniques such as decision trees and ensemble methods demonstrated improved performance by leveraging the inherent structure of financial data (8). However, these approaches often required extensive preprocessing and feature selection, which demanded significant domain expertise. while these models enhanced predictive accuracy, their complexity sometimes hindered interpretability, making it challenging for stakeholders to fully trust the outcomes. Nonetheless, these algorithmic advancements marked a pivotal shift toward more flexible and data centric approaches in financial risk modeling (9).

Building on these developments, the integration of advanced neural network architectures has revolutionized predictive modeling in corporate financial risk. These models, characterized by their ability to learn hierarchical representations from raw data, have significantly improved the accuracy and scalability of risk assessments. Deep learning techniques, including recurrent and convolutional networks, excel at capturing intricate temporal and spatial patterns within financial datasets (10). Furthermore, the adoption of transfer learning and pre trained models has enabled researchers to leverage existing knowledge for specialized tasks, reducing the need for extensive data collection. Despite their remarkable capabilities, these methods face challenges such as high computational demands and limited transparency in decision making processes. Nevertheless, the advancements in neural network based modeling continue to push the boundaries of predictive accuracy and offer promising avenues for addressing complex financial risk scenarios (11).

Based on the limitations of the aforementioned methods, we propose a novel approach to predictive modeling of corporate financial risk that addresses these challenges. By combining structured knowledge representation with advanced data driven techniques, our approach offers a more holistic view of financial risk factors. This integration allows for the incorporation of expert knowledge while leveraging the power of machine learning and deep learning to adapt to dynamic market conditions. Furthermore, our method emphasizes transparency and interpretability, ensuring that stakeholders can understand and trust the predictions generated by the model. By addressing the limitations of previous approaches, our method aims to provide a robust solution for predicting corporate financial risk and its implications for health economics and public health efficiency.

We summarize our contributions as follows:

- We propose a unified conceptual and analytical framework that links corporate financial risk to health economics and public health efficiency through clearly defined transmission mechanisms.
- We develop a predictive modeling approach that integrates probabilistic inference, temporal dynamics, and multimodal data to capture complex and uncertain relationships between financial indicators and health system outcomes.
- We provide empirical evidence demonstrating that the proposed framework improves predictive performance while offering interpretable insights that support decision making in health economics and public policy contexts.

Although the present study employs predictive modeling tools, its substantive focus is not on algorithmic innovation alone. Rather, the paper is motivated by a cross disciplinary economic question: how firm level financial instability may be associated with expenditure pressure, service disruption, and efficiency loss in health related systems through organizational, labor market, insurance, and fiscal transmission channels. In this sense, the modeling framework is intended as an analytical instrument for examining a financially and economically relevant cross sector relationship, rather than as a purely computer science or management oriented prediction exercise.

## 2 Related work

### 2.1 Financial risk prediction models

The development of predictive models for corporate financial risk has long been a central topic in finance and applied economics. Early studies primarily relied on statistical approaches, such as regression based models and financial ratio analysis, to estimate the likelihood of bankruptcy or financial distress. These methods provided interpretable frameworks but were often limited in their ability to capture complex, non-linear relationships in financial data (12). Subsequent research introduced machine learning techniques, including decision trees, ensemble models, and neural networks, which significantly improved predictive performance by exploiting high dimensional data and non-linear dependencies. More recent studies have further incorporated deep learning architectures and data driven approaches to enhance scalability and accuracy, particularly in dynamic and uncertain financial environments (13). Despite these advancements, existing financial risk prediction models are predominantly designed for firm level outcomes, such as default probability or credit risk, and typically do not account for the broader economic and societal implications of financial instability. In particular, the linkage between corporate financial risk and health system performance remains largely unexplored. This limitation motivates the need for modeling frameworks that extend beyond firm level prediction and incorporate cross sectoral impacts, especially in domains such as health economics (14).

### 2.2 Impact on health economics

Health economics research has extensively examined how financial constraints influence healthcare systems, focusing on resource allocation, cost efficiency, and access to services. A substantial body of work highlights that budgetary pressures and financial instability can reduce healthcare investment, delay service provision, and constrain the adoption of new technologies, thereby affecting both efficiency and equity in healthcare delivery (15). Another important strand of literature investigates how socioeconomic conditions, including employment status, income stability, and insurance coverage, shape healthcare utilization and population health outcomes. These studies emphasize that economic shocks can propagate through multiple channels, ultimately influencing both the demand for and supply of healthcare services (16). However, the majority of existing studies treat financial conditions as macroeconomic or sector level variables and rarely incorporate firm level financial risk as a driving factor. As a result, the micro to macro transmission mechanisms linking corporate financial instability to health system performance are insufficiently addressed. This gap highlights the need for an integrated perspective that connects firm level financial dynamics with system level health economic outcomes (17).

### 2.3 Public health efficiency strategies

Improving public health efficiency has been a major objective in both health policy and applied research. Public health efficiency is commonly understood as the ability of health systems to deliver high quality services while optimizing the use of limited resources. Existing strategies focus on financial management practices, resource allocation optimization, and the adoption of digital health technologies to enhance service delivery and reduce costs (18). Recent studies have emphasized the role of data analytics and decision support systems in improving operational efficiency, enabling policymakers and administrators to respond more effectively to changing demand and resource constraints. Institutional coordination and policy design have been identified as critical factors for maintaining system resilience under financial pressure (19). Nevertheless, current approaches to public health efficiency are largely reactive and descriptive, relying on observed indicators rather than forward looking risk assessments. The integration of predictive financial risk modeling into public health planning remains limited. In particular, there is a lack of frameworks that can anticipate how financial instability in the corporate sector may translate into efficiency losses in health systems (20).

While prior research has made substantial progress in financial risk prediction, health economics, and public health efficiency as separate domains, there is a clear lack of integrated frameworks that jointly model the transmission of corporate financial risk to health system outcomes. This study addresses this gap by developing a unified modeling approach that combines financial risk prediction with health economic analysis, enabling a more comprehensive understanding of cross sectoral risk propagation. Taken together, the relevant literature for this study lies not only in predictive risk modeling, but also in the economics and finance literature on financial distress, resource allocation, macroeconomic spillovers, and health system efficiency under fiscal constraint.

## 3 Method

### 3.1 Overview

This section presents the methodological framework developed for predictive modeling of corporate financial risk and its implications for health economics and public health efficiency. The primary objective is to construct a robust system capable of forecasting corporate financial risk while capturing its broader economic and health system consequences. To this end, we begin by formalizing the problem through a set of mathematical and theoretical constructs that characterize the dynamics of financial risk and its cascading effects on health economic outcomes. Key variables, parameters, and relationships are defined to establish a coherent predictive framework, providing the foundation for modeling the complex interactions between financial indicators and system level health performance.

Building on this formulation, we introduce the proposed predictive model, termed the Risk Economic Health Nexus Model (REHNM), which integrates heterogeneous data sources and leverages data driven techniques to capture the relationship between corporate financial stability and public health metrics. To facilitate practical application, we further develop the Strategic Risk Health Integration Approach (SRHIA), which translates model outputs into actionable insights for policymakers and health economists. The selection of modeling techniques is guided by the characteristics of the research problem rather than by methodological complexity alone. The present research question is not limited to a static firm level classification task. Instead, it concerns a structured predictive problem in which corporate financial conditions may belong to heterogeneous risk regimes, their implications for health related outcomes are mediated by partially unobserved organizational and socioeconomic processes, and these relationships may evolve over time and vary across macroeconomic and policy contexts. For this reason, probabilistic modeling is adopted to represent uncertainty and heterogeneous risk states, latent variable structures are used to capture intermediate transmission conditions that are not directly observed, temporal modeling is introduced to reflect persistence and transition in financial stress, and exogenous variables are included to account for contextual modifiers of risk transmission. Simpler predictive models remain useful as benchmarks and are therefore included in the comparative experiments, but they are less well-suited to representing these joint features of heterogeneity, mediation, temporal evolution, and contextual dependence within a unified framework. In particular, the framework is designed to address three key challenges: heterogeneity in financial–health relationships, uncertainty arising from incomplete information and unobserved transmission mechanisms, and temporal dynamics driven by evolving economic conditions. Probabilistic modeling is employed to represent uncertainty, latent variable structures are introduced to capture unobserved intermediate mechanisms consistent with the conceptual pathways outlined in the Introduction, and temporal modeling is incorporated to reflect the evolution of financial risk over time. These modeling choices are particularly suitable for the dual structure of the present study. On the financial risk side, corporate distress is heterogeneous across firms and evolves over time under changing market and policy conditions; therefore, probabilistic and temporal components are needed to capture multiple risk regimes, persistence, and transition. On the health related outcome side, the consequences of financial instability are multidimensional and only partially observable, spanning expenditure pressure, service accessibility, continuity of provision, and efficiency of resource use; therefore, latent variable and multimodal components are needed to represent intermediate transmission conditions and the joint variation of multiple outcome dimensions. In this way, the selected modeling framework is designed to align with both the dynamic nature of financial risk and the structured, system level nature of the health related outcomes considered in this study.

Exogenous variables are included to account for policy, macroeconomic, and structural factors that may influence both financial risk and health system performance. More advanced components, such as multiple hypothesis prediction and adversarial scenario generation, are incorporated as auxiliary mechanisms to enhance robustness, represent multiple plausible future scenarios, and support stress testing under uncertainty, rather than as standalone modeling objectives. The proposed framework provides an integrated approach for analyzing the interaction between corporate financial risk and public health efficiency, offering both predictive capability and decision support value in complex and uncertain environments. For readers from public health and policy backgrounds, the methodological framework can be interpreted in simpler terms as a structured early warning system with three core functions: identifying different patterns of corporate financial stress, tracking how those patterns evolve over time, and assessing how they may be associated with pressures on health expenditure, service continuity, and public health efficiency. The technical components are introduced to support these substantive goals, rather than to increase methodological complexity for its own sake. This perspective may help situate the modeling framework within the broader concerns of health system planning, risk monitoring, and policy interpretation.

The novelty of the present study should therefore be understood at the level of problem formulation and framework integration rather than as the introduction of an entirely isolated prediction algorithm. Existing financial risk prediction models in financial econometrics and machine learning are primarily designed for firm level outcomes such as distress, bankruptcy, or credit risk, whereas health economics models typically examine expenditure, access, or efficiency from macroeconomic, institutional, or system level determinants. By contrast, the present study proposes a cross domain framework that links firm level financial instability to multidimensional health system outcomes through intermediate transmission mechanisms. Within this architecture, REHNM serves as the unified analytical core for modeling the structured association between financial conditions and health related outcomes, DRAM extends this core by capturing temporal evolution and contextual modulation of risk transmission, and SRHIA translates predictive outputs into policy relevant interpretation and scenario based decision support. Accordingly, the contribution of the manuscript lies not merely in renaming existing techniques, but in integrating established probabilistic, dynamic, and multimodal modeling elements into a theoretically structured framework for analyzing the financial health nexus.

### 3.2 Preliminaries

This section formalizes the predictive modeling problem linking corporate financial risk to health economics and public health efficiency. Let X denote the vector space of corporate financial indicators and Y the space of health related outcomes. The relationship between these variables is represented by a joint distribution *p*(*x, y*), where x∈X captures firm level financial conditions and y∈Y represents system level health and economic outcomes.

In this study, the outcome space Y is defined as a multidimensional representation of health system consequences rather than a single clinical endpoint. It includes health economic indicators, public health service indicators, and public health efficiency measures reflecting how effectively financial and institutional resources are translated into service provision. The mapping from X to Y is therefore interpreted as capturing transmission through intermediate organizational and socioeconomic mechanisms, such as financing constraints, labor market spillovers, and disruptions in service provision. Accordingly, the model is designed to capture predictive and structural associations, while avoiding strong causal claims in the absence of an explicit identification strategy. The empirical objective of this study is to identify and predict structured associations between corporate financial conditions and health related system outcomes, not to establish causal effects in the econometric sense. Any causal interpretation would require additional identification strategies, such as exogenous variation, quasi experimental designs, or stronger institutional assumptions, which are beyond the scope of the present analysis.

The predictive task is to learn a function $f_{\theta }:X\to Y$ that minimizes the expected loss over observed data ${\left\{\left(x_{i},y_{i}\right)\right\}}_{i=1}^{N}$ (Equation 1):


$$\begin{array}{l} {𝔼}_{\left(x,y\right)\sim p\left(x,y\right)}\left[L\left(f_{\theta }\left(x\right),y\right)\right], \end{array}$$ (1)

where *L*(·, ·) measures the discrepancy between predicted and observed outcomes. To account for uncertainty, we model the conditional distribution *p*(*y*|*x*) as Equation 2:


$$\begin{array}{l} p\left(y|x\right)={\text{∫}}_{\text{Θ}}p\left(y|x,\theta \right)p\left(\theta \right)d\theta , \end{array}$$ (2)

where *p*(θ) represents the prior over parameters. Furthermore, a latent variable z∈Z is introduced to capture unobserved factors influencing both financial risk and health outcomes (Equation 3):


$$\begin{array}{l} p\left(x,y,z\right)=p\left(y|x,z\right)p\left(x|z\right)p\left(z\right). \end{array}$$ (3)

From a substantive perspective, *z* can be interpreted as representing meso level transmission conditions, including labor market stress, fiscal pressure, insurance instability, and disruptions in healthcare supply chains. This formulation reflects the idea that the relationship between firm level risk and system level outcomes is mediated by intermediate structural processes rather than being a direct one step effect.

The model is estimated using a dataset $D={\left\{\left(x_{i},y_{i}\right)\right\}}_{i=1}^{N}$ by maximizing the likelihood (Equation 4):


$$\begin{array}{l} \theta^{*}=\arg\underset{\theta }{\max}\overset{N}{\underset{i=1}{\sum }}\log p\left(y_{i}|x_{i},\theta \right). \end{array}$$ (4)

This probabilistic framework allows the model to capture uncertainty and hidden dependencies, providing a theoretically grounded basis for the predictive and analytical methods developed in subsequent sections.

### 3.3 Dynamic Risk Assessment Model

In this subsection, we introduce the Dynamic Risk Assessment Model (DRAM), which serves as the dynamic modeling component of the broader Risk Economic Health Nexus framework. The purpose of DRAM is not only to estimate corporate financial risk at a given point in time, but also to characterize how such risk evolves under changing economic, policy, and institutional conditions, and how this evolving risk may translate into pressure on health economic and public health efficiency outcomes. In this study, DRAM is therefore used to bridge firm level financial signals and system level health consequences through an intermediate dynamic risk state. DRAM models the relationship between corporate financial indicators and a latent risk score *R*, where *R* is interpreted as a composite representation of financial stress with potential downstream implications for healthcare expenditure pressure, service accessibility, continuity of provision, and resource use efficiency. This formulation is intended to capture predictive and structural associations rather than strict causal effects. The model is designed to address three empirical challenges: heterogeneity across financial risk scenarios, temporal evolution in financial stress, and sensitivity to exogenous contextual factors.

**Figure 1:**
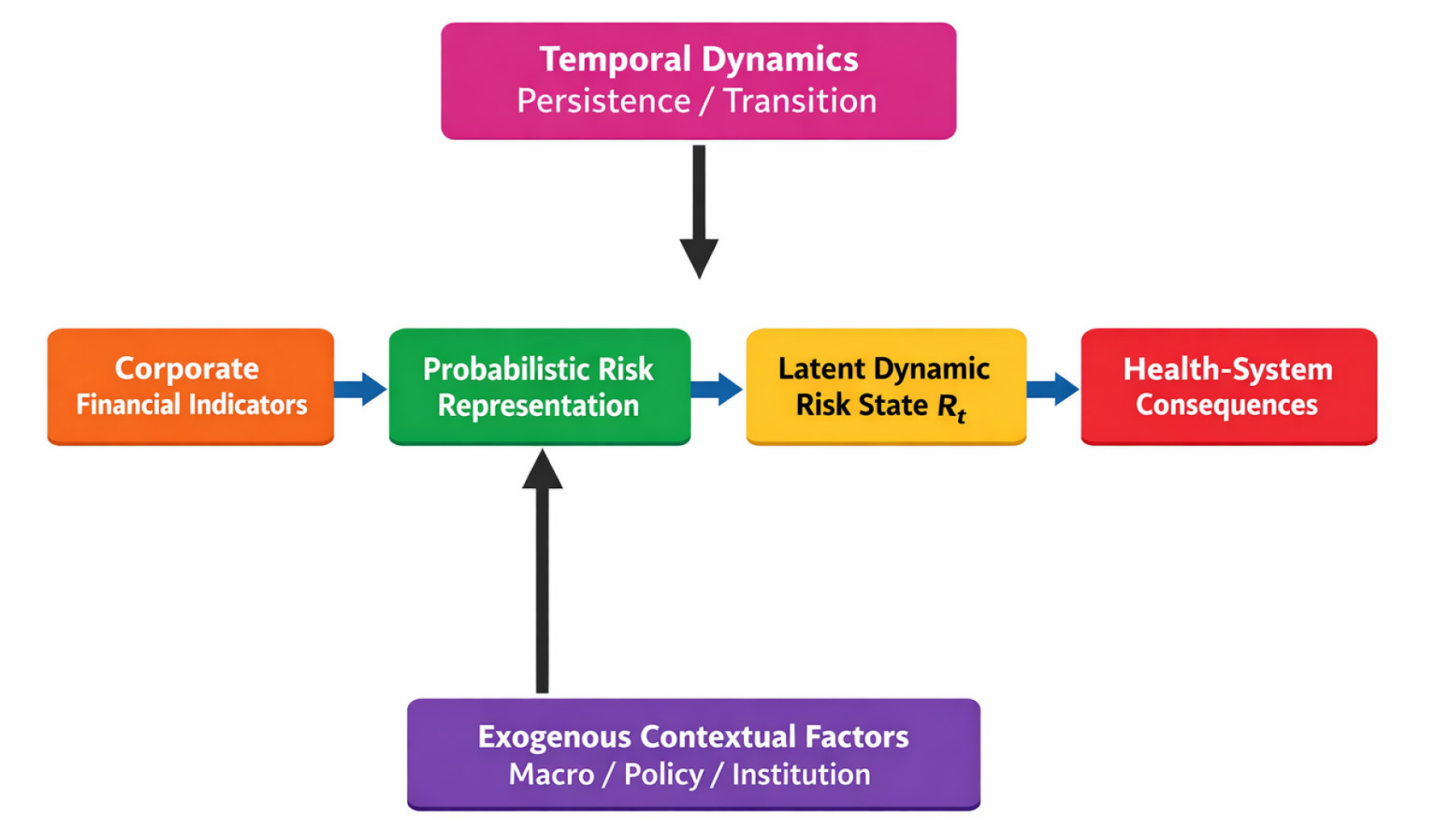
Conceptual architecture of the Dynamic Risk Assessment Model (DRAM). Firm level financial indicators are first organized into a multidimensional probabilistic risk representation, from which a latent dynamic risk state *R*_*t*_ is estimated. This intermediate risk state captures evolving financial stress and serves as the transmission layer linking corporate financial instability to health system consequences, including expenditure pressure, service accessibility, continuity of provision, and public health efficiency. Temporal dynamics and exogenous contextual factors act as conditioning mechanisms that shape the evolution and impact of the risk state.

Figure 1 provides a schematic overview of the role of DRAM within the proposed framework. The figure is intended to clarify that DRAM does not merely generate a static prediction of firm level financial risk; rather, it organizes financial indicators into a dynamic risk representation that evolves over time and is conditioned by external contextual factors. This intermediate dynamic risk state is then used to interpret how corporate financial instability may be associated with downstream pressures on health economics and public health efficiency. As illustrated in Figure 1, the DRAM framework consists of three tightly connected components. The first component identifies heterogeneous financial risk regimes through a probabilistic representation of firm level indicators. The second introduces temporal dependence to capture the persistence and evolution of financial stress. The third incorporates exogenous variables that modify the way financial risk is transmitted to health related system outcomes. The following subsections describe these components in turn.

#### 3.3.1 Multidimensional probabilistic framework

We first represent the financial risk landscape as a multidimensional feature space, where each dimension corresponds to a distinct corporate financial indicator. Let **X** = {*x*_1_, *x*_2_, …, *x*_*n*_} denote the observed financial indicators, and let *R* denote the latent risk score summarizing the potential health system relevance of corporate financial instability. The mapping from **X** to *R* is defined through a function *f*:ℝ^*n*^ → ℝ, such that Equation 5:


$$\begin{array}{l} R=f\left(\text{X}\right). \end{array}$$ (5)

To accommodate heterogeneity and uncertainty in financial states, we model the distribution of **X** using a Gaussian Mixture Model (GMM; Equation 6):


$$\begin{array}{l} p\left(\text{X}|\text{Θ}\right)=\overset{K}{\underset{k=1}{\sum }}\pi_{k}N\left(\text{X}|\mu_{k},\Sigma_{k}\right), \end{array}$$ (6)

where *K* is the number of mixture components, π_*k*_ is the mixing coefficient, and μ_*k*_ and Σ_*k*_ are the mean vector and covariance matrix of the *k*-th component, respectively. The parameter set is given by $\text{Θ}={\left\{\pi_{k},\mu_{k},\Sigma_{k}\right\}}_{k=1}^{K}$. Under this formulation, each mixture component can be interpreted as a distinct financial risk regime, allowing the model to represent multiple plausible corporate conditions rather than imposing a single homogeneous risk structure. The expected risk score is then computed as Equation 7:


$$\begin{array}{l} R=\overset{K}{\underset{k=1}{\sum }}\pi_{k}𝔼\left[f\left(\text{X}\right)|\mu_{k},\Sigma_{k}\right], \end{array}$$ (7)

which yields a weighted summary of risk across alternative financial states.

#### 3.3.2 Temporal dynamics integration

Because corporate financial stress is not static, DRAM further incorporates temporal dependence in order to capture the evolution of risk over time. Let **X**_*t*_ denote the vector of financial indicators at time *t*. We assume that the transition of financial conditions follows a first order stochastic process (Equation 8):


$$\begin{array}{l} p\left({\text{X}}_{t+1}|{\text{X}}_{t}\right)=N\left({\text{X}}_{t+1}|\text{A}{\text{X}}_{t}+\text{b},\text{Q}\right), \end{array}$$ (8)

where **A** is the transition matrix, **b** is the offset vector, and **Q** is the covariance matrix of process noise. This specification enables the model to capture persistence, fluctuation, and transition in firm level financial conditions. From the perspective of health economics, temporal modeling is important because the effect of financial distress on expenditure pressure, service continuity, and public health efficiency is often cumulative and delayed rather than instantaneous. By incorporating this dynamic structure, DRAM provides a more realistic representation of the way financial instability propagates across periods.

**Figure 2:**
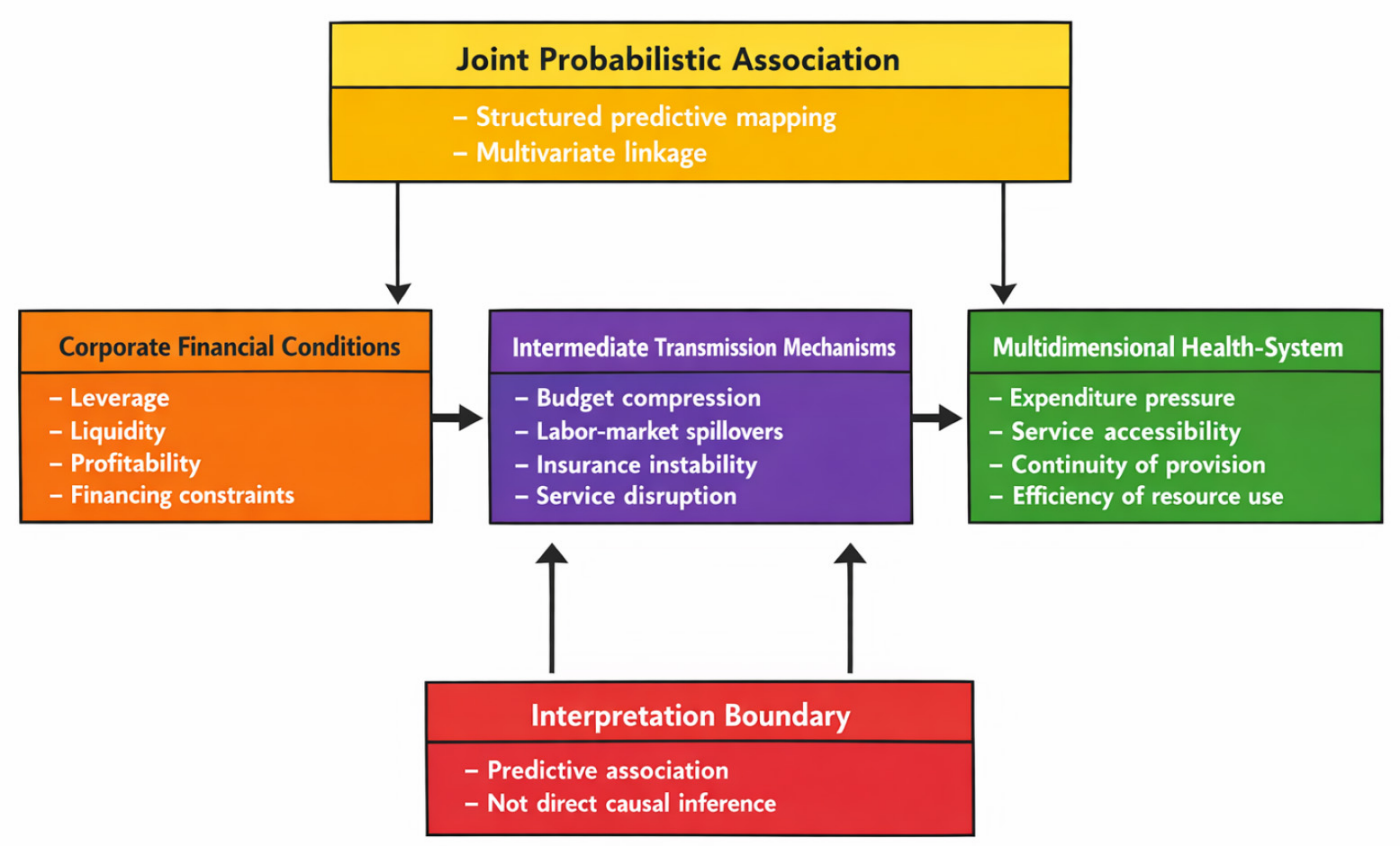
Analytical mapping from corporate financial conditions to multidimensional health system outcomes. Firm level financial conditions are linked to expenditure pressure, service accessibility, continuity of provision, and efficiency related outcomes through intermediate transmission mechanisms such as budget compression, labor market spillovers, insurance instability, and service disruption. The figure emphasizes that the framework estimates structured predictive associations rather than direct causal effects.

While Figure 1 emphasizes the internal structure of DRAM, Figure 2 highlights the analytical mapping that connects corporate financial conditions to health system consequences. This second diagram is useful for clarifying the substantive interpretation of the model, namely that financial indicators are not linked directly to a single health endpoint, but rather to a multidimensional outcome space that reflects expenditure pressure, service accessibility, continuity of provision, and efficiency of resource utilization. As shown in Figure 2, the proposed framework is structured around a probabilistic association between financial conditions and health related outcomes, with the latent risk state serving as the key transmission layer. This representation is consistent with the conceptual discussion in the Introduction and the formal definitions in Section 3.2, where the relationship of interest is interpreted as a structured predictive association mediated by intermediate organizational and socioeconomic mechanisms. This perspective motivates the inclusion of exogenous contextual variables in the final component of DRAM.

#### 3.3.3 Exogenous variable integration

To endogenous financial indicators, the model incorporates contextual variables that may alter the strength or direction of risk transmission. Let **Z** = {*z*_1_, *z*_2_, …, *z*_*m*_} denote exogenous variables, including macroeconomic conditions, policy interventions, regulatory changes, or broad health system shocks. Their contribution to the latent risk score is modeled as Equation 9:


$$\begin{array}{l} R=\beta_{0}+\overset{m}{\underset{j=1}{\sum }}\beta_{j}z_{j}+\epsilon , \end{array}$$ (9)

where β_*j*_ are regression coefficients and ϵ is an error term. This component allows DRAM to account for the fact that similar firm level financial conditions may generate different downstream consequences depending on the surrounding policy and institutional environment. In substantive terms, the exogenous module captures how corporate financial risk is filtered through macroeconomic pressure, public financing arrangements, and system resilience capacity before being reflected in health related outcomes.

Taken together, the multidimensional probabilistic structure, temporal dynamics, and exogenous adjustment make DRAM a flexible tool for analyzing how corporate financial instability may evolve and how it may be associated with health economic and public health efficiency pressures. In the broader framework of this study, DRAM functions as an intermediate analytical layer that supports forecasting, scenario analysis, and stress testing, while also improving the interpretability of the transmission from firm level risk exposure to system level consequences.

### 3.4 Innovative risk mitigation strategy

Building on the predictive and dynamic modeling components introduced in the previous sections, this subsection presents the risk mitigation and decision support layer of the proposed framework. The purpose of this component is not to introduce an entirely separate prediction model, but to translate estimated financial risk into actionable information for policy analysis, scenario evaluation, and resilience oriented planning in health economics and public health systems. In this sense, the strategy complements the DRAM by focusing on how predicted risk can be interpreted, stress tested, and operationalized under uncertainty.

**Figure 3:**
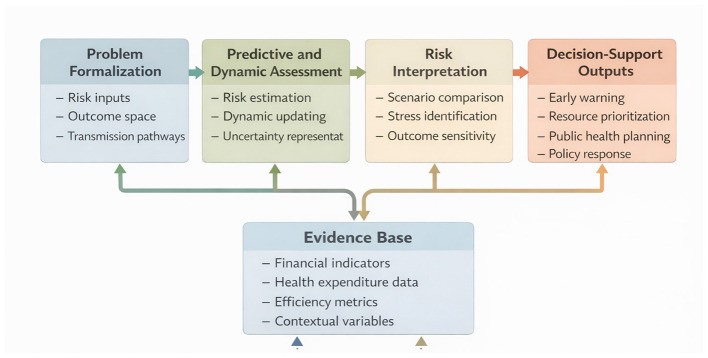
Overall logic of the risk mitigation and decision support layer. The framework proceeds from problem formalization to predictive and dynamic assessment, followed by risk interpretation and policy oriented outputs. Multi source evidence, including financial indicators, health expenditure data, efficiency metrics, and contextual variables, supports the translation of estimated financial risk into early warning, resource prioritization, public health planning, and resilience oriented policy response.

Figure 3 summarizes the overall logic of this mitigation oriented framework. The figure illustrates how problem formalization, predictive modeling, and interdisciplinary interpretation are linked in sequence, so that firm level financial risk signals can be transformed into structured assessments of health system pressure and policy relevance. Rather than treating financial prediction as an end in itself, the framework positions prediction as an input into a broader process of risk interpretation and mitigation.

As illustrated in Figure 3, this strategy is organized around three complementary functions: multimodal predictive integration, adaptive dependency modeling, and scenario based stress testing. Together, these functions allow the framework to move beyond point prediction and support a more policy relevant analysis of how corporate financial instability may affect health system expenditure pressure, service continuity, and operationa efficiency.

#### 3.4.1 Multimodal predictive integration

The first component integrates heterogeneous sources of information, including corporate financial indicators, market conditions, and health related system variables, into a unified predictive structure. Let *X* denote the space of financial indicators and *Y* the space of health related outcomes. Their joint relationship is represented as Equation 10:


$$\begin{array}{l} p\left(x,y\right)=p\left(y|x\right)p\left(x\right), \end{array}$$ (10)

where *p*(*y*∣*x*) characterizes the conditional association between financial conditions and health system consequences. The predictive objective is to learn a function *f*_θ_:*X*→*Y* that minimizes the expected loss (Equation 11):


$$\begin{array}{l} {𝔼}_{\left(x,y\right)\sim p\left(x,y\right)}\left[L\left(f_{\theta }\left(x\right),y\right)\right]. \end{array}$$ (11)

Within this structure, multiple hypothesis prediction is used as an auxiliary device to represent uncertainty by generating several plausible outcome trajectories rather than a single deterministic forecast. This is particularly relevant when financial shocks may propagate through different institutional and socioeconomic channels, leading to multiple feasible health system responses.

**Figure 4:**
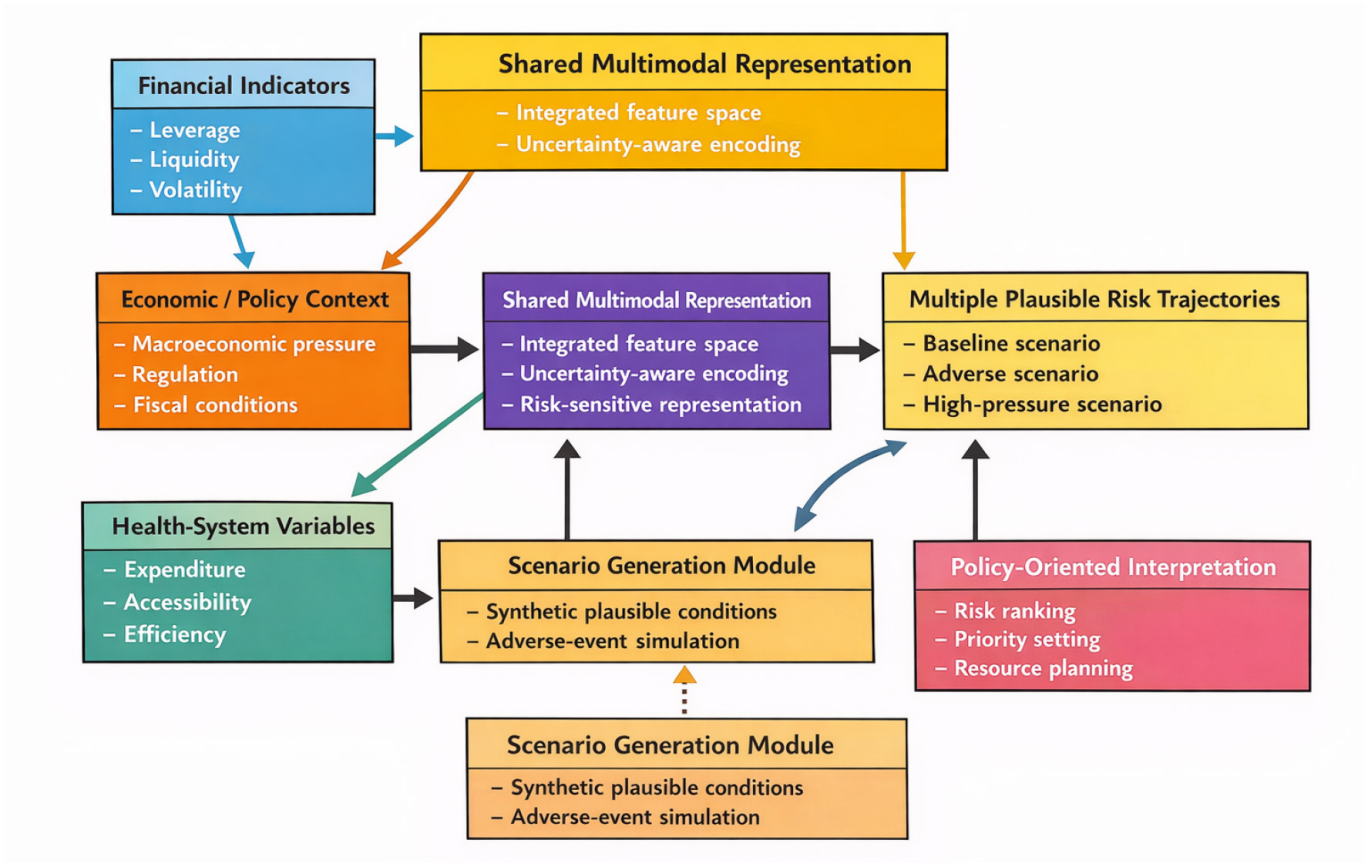
Operational flow of the mitigation oriented predictive framework. Financial indicators, economic and policy context, and health system variables are integrated into a shared multimodal representation, from which multiple plausible risk trajectories are generated. These trajectories support stress testing under adverse conditions and policy oriented interpretation for risk ranking, priority setting, and resource planning. A scenario generation module is included to simulate plausible disruptive conditions for robustness assessment.

To make this predictive structure operational, Figure 4 illustrates the flow of information across the different data streams and analytical components. The figure emphasizes that the framework combines financial, economic, and health related inputs in order to generate scenario sensitive predictions that can subsequently support risk mitigation and policy interpretation.

As shown in Figure 4, the model is designed to combine heterogeneous information into a shared representation that remains sensitive to uncertainty and contextual variation. This architecture supports the transition from raw financial signals to interpretable health system risk profiles, thereby improving the practical usefulness of the framework for decision makers.

#### 3.4.2 Adaptive dependency modeling

A second component of the strategy is designed to capture complex and context dependent relationships among financial indicators and health related variables. Instead of assuming fixed dependencies, the framework allows the interaction structure to adapt to changing conditions. This adaptive mechanism is especially important in settings where the effect of corporate financial stress may differ across institutional environments, regulatory contexts, or stages of economic disruption. In methodological terms, this component functions as a flexible dependency learning layer that improves the model's ability to capture heterogeneous transmission patterns while preserving the broader substantive interpretation of the framework.

#### 3.4.3 Scenario generation for stress testing

The third component introduces a scenario generation mechanism for evaluating model robustness under adverse or uncertain conditions. Rather than treating observed data as the only relevant environment, this module generates synthetic but plausible financial stress scenarios that can be used to assess how predicted risks and health system pressures may change under alternative macroeconomic or institutional conditions. Formally, this mechanism follows an adversarial setup (Equation 12):


$$\begin{array}{l} \underset{G}{\min}\underset{D}{\max}{𝔼}_{x\sim p_{\text{data}}\left(x\right)}\left[\log D\left(x\right)\right]+{𝔼}_{z\sim p_{z}\left(z\right)}\left[\log\left(1-D\left(G\left(z\right)\right)\right)\right], \end{array}$$ (12)

where *G* generates candidate scenarios and *D* evaluates their plausibility relative to observed data. In the present framework, the role of this component is not to replace the core predictive model, but to support stress testing and scenario analysis. This helps identify conditions under which financial instability may exert stronger pressure on healthcare expenditure, service accessibility, or public health efficiency.

The innovative risk mitigation strategy extends the proposed framework from prediction to application. By integrating multimodal information, adaptive dependency modeling, and scenario based stress testing, it provides a structured way to convert estimates of corporate financial risk into decision support tools for public health planning, resource prioritization, and resilience oriented health economic analysis.

## 4 Experimental setup

### 4.1 Dataset

To evaluate the proposed framework, this study integrates four complementary data sources capturing different dimensions of the relationship between corporate financial risk and health system outcomes. These sources were selected to reflect the multidimensional structure of the problem, combining firm level financial indicators, health economic expenditure patterns, public health efficiency measures, and broader socioeconomic variables associated with health outcomes. In the context of the present study, the financial datasets provide the main inputs for risk estimation, whereas the health related datasets provide the outcome dimensions used to assess the downstream relevance of corporate financial instability.

The first data category, referred to here as corporate financial risk indicators, contains firm level variables commonly used in financial distress and risk prediction, including leverage measures, liquidity ratios, credit related indicators, profitability metrics, and market volatility proxies. These variables form the primary feature space used to represent corporate financial conditions in the model. In substantive terms, this dataset captures the intensity and composition of financial stress across firms and sectors, thereby serving as the core input for estimating the latent financial risk state introduced in the methodological sections. The second and third data categories provide the health related outcome dimensions of the study. The Health Economics Expenditure Trends Dataset includes indicators related to healthcare spending, such as public and private expenditure, insurance related financial burden, and out of pocket costs. These variables are used to represent expenditure pressure and cost dynamics within health systems. The Public Health Efficiency Metrics Dataset complements this by including measures associated with service accessibility, delivery capacity, timeliness, and resource use efficiency. Together, these two datasets operationalize the outcome space of the model by reflecting both the economic burden placed on health systems and the efficiency with which available resources are converted into public health services.

In operational terms, health economics in this study is measured through expenditure oriented indicators that reflect financial pressure within health systems, including current health expenditure, public and private spending shares, out of pocket burden, and insurance related cost pressure. Public health efficiency is measured through service oriented and resource use indicators, including accessibility of services, delivery capacity, timeliness, continuity of provision, and proxy measures of how effectively available resources are translated into health services. Rather than relying on a single proxy, both constructs are treated as multidimensional outcome domains represented by complementary indicators drawn from established public databases. The validity of these measurements is supported in three ways. The indicators are drawn from widely used international data sources, including WHO, World Bank, and OECD repositories, whose definitions are commonly used in health economics and health system research. The selected indicators are aligned with the conceptual framework developed in this study, which distinguishes expenditure pressure from service efficiency as two related but non-identical dimensions of system level impact. The consistency of the empirical results across comparative experiments and ablation analyses provides indirect support that these outcome definitions capture stable and interpretable patterns rather than arbitrary measurement noise.

The fourth data category, referred to as financial risk and health related contextual indicators, includes broader socioeconomic and health related variables such as employment conditions, income related vulnerability, access to healthcare, and selected health status indicators. In the present study, this dataset is not treated as a direct causal identification tool, but rather as a supplementary source for examining whether the estimated financial risk patterns are associated with broader health relevant conditions. It therefore serves as a complementary layer for evaluating the external relevance of the proposed framework.

To construct the analytical dataset, variables from these sources are harmonized through indicator matching, temporal alignment, and standard preprocessing procedures. Continuous variables are normalized where appropriate, missing observations are handled through consistent cleaning and imputation rules, and indicators are grouped into financial, expenditure, service, and efficiency dimensions before model estimation. This integration process enables the framework to connect firm level financial conditions with system level health related outcomes while preserving the multidimensional structure of the research question. At the same time, several limitations should be acknowledged. The integrated dataset may be affected by differences in reporting standards across sources, potential measurement error in health system indicators, and incomplete observation of intermediate transmission mechanisms. For this reason, the empirical analysis is interpreted as evidence of structured predictive association rather than definitive causal identification. Table 1 summarizes the four data categories used in this study, the representative variables compiled from multiple public sources within each category, and their analytical roles in the proposed framework. This organization clarifies how firm level financial indicators are linked to system level health expenditure, public health efficiency, and broader socioeconomic conditions through a harmonized multi source dataset.

**Table 1:** Summary of data sources, variables, and analytical roles in the proposed framework.

Data source category	Representative variables	Role in this study	Primary public source(s)	Data level
Corporate financial risk indicators	Leverage, liquidity, profitability, credit constraints, sales performance, firm characteristics	Main input space *X* for estimating firm level financial stress and latent risk states	World Bank Enterprise Surveys; firm level financial reporting and business environment indicators	Firm/enterprise
Health economics expenditure indicators	Current health expenditure, public/private spending, out of pocket expenditure, insurance related burden	Outcome dimension of *Y* capturing expenditure pressure and cost dynamics	WHO Global Health Expenditure Database (GHED); World Bank WDI health expenditure indicators	Country/system
Public health efficiency indicators	Service accessibility, health system resources, selected quality/access indicators, timeliness, system performance proxies	Outcome dimension of *Y* capturing service delivery capacity and efficiency of resource use	OECD Health Statistics/Health at a Glance; WHO Global Health Observatory; World Bank WDI	Country/system
Financial risk and health related contextual indicators	Employment, labor market stress, income vulnerability, selected health status indicators, access related measures	Supplementary contextual layer for examining broader associations and transmission conditions	ILOSTAT; World Bank WDI; WHO Global Health Observatory	Country/population

The selection of data sources and variables was guided by three principles: conceptual relevance to the proposed transmission framework, frequent use in the related literature, and cross source availability for harmonized empirical analysis. Variables were retained only if they could be meaningfully assigned to one of the four analytical dimensions of the study, namely firm level financial stress, health expenditure pressure, public health service efficiency, or broader socioeconomic and health related context. For the corporate financial risk dimension, we prioritized indicators that are commonly used to characterize solvency, liquidity, profitability, operating performance, and financing constraints. For the health economics and public health dimensions, we retained indicators that capture expenditure burden, service accessibility, continuity of provision, delivery capacity, and efficiency related system performance. Contextual indicators were selected to reflect labor market conditions, income vulnerability, insurance related instability, and access related health conditions that may mediate or condition the relationship of interest. To construct the final analytical dataset, variables from these public sources were matched at the closest feasible common unit of analysis after temporal alignment and indicator harmonization. Variables with excessive missingness, weak comparability across sources, or ambiguous conceptual roles in the transmission framework were excluded. Continuous variables were standardized after cleaning, and the retained indicators were organized into input, outcome, and contextual groups for model estimation. This procedure was intended to balance theoretical interpretability, empirical coverage, and cross source consistency in a setting where firm level and system level information cannot always be perfectly aligned.

All datasets used in this study are based on publicly accessible secondary data sources rather than on simulated data or proprietary private databases. The empirical analysis draws on established international repositories, including the World Bank, WHO, OECD, and ILOSTAT, from which the relevant firm level, health expenditure, public health efficiency, and contextual indicators were obtained. The final analytical dataset used for model estimation is therefore not a single pre packaged benchmark dataset, but a harmonized multi source dataset constructed by the author through indicator matching, temporal alignment, cleaning, and standardization across these public sources. The reliability of these data is supported by the fact that the underlying indicators are drawn from widely used international databases with standardized documentation and broad use in empirical research. Their representativeness was considered in relation to the analytical purpose of the study: firm level indicators were used to characterize corporate financial stress, while country and system level indicators were used to reflect expenditure pressure, service accessibility, and public health efficiency. At the same time, we do not claim perfect coverage or measurement equivalence across all sources. For this reason, the integrated dataset should be understood as a theoretically guided and empirically harmonized representation of the financial health nexus, rather than as an exhaustive or causally identified population dataset.

### 4.2 Experimental details

This subsection describes the implementation and evaluation procedures used to assess the proposed framework. All experiments were implemented in PyTorch (PyTorch Foundation/The Linux Foundation, Wilmington, Delaware, USA) and conducted on a workstation equipped with NVIDIA Tesla V100 GPUs (NVIDIA Corporation, Santa Clara, California, USA). The computational setting was selected to ensure stable model training and efficient handling of the integrated multi source dataset. In line with the objectives of this study, the experimental design emphasizes reproducibility, comparability across models, and consistency with the structured financial and health related data used in the analysis. Taken together, the main contributions of this study can be summarized in three aspects. We develop a unified conceptual framework that explains how firm level financial risk may be linked to health economics and public health efficiency through direct, indirect, and systemic transmission mechanisms. We propose an integrated predictive modeling framework that combines probabilistic representation, temporal dynamics, and contextual adjustment to analyze this cross domain relationship under uncertainty. We show through comparative and ablation analyses that the proposed framework provides improved predictive performance and can serve as a structured decision support tool for early warning, policy interpretation, and resilience oriented planning.

Before model estimation, variables from the four data source categories were harmonized through temporal alignment, indicator matching, and standard preprocessing procedures. Continuous variables were standardized to reduce scale heterogeneity across financial, expenditure, service, and socioeconomic indicators. Missing observations were handled through a consistent cleaning and imputation pipeline, and variables with excessive incompleteness were excluded from the final analytical sample. We note, however, that these preprocessing steps do not fully eliminate potential data limitations. In particular, because the empirical analysis relies on multiple public sources collected under different reporting conventions, some degree of measurement error and residual comparability bias may remain. Similarly, although missing observations were treated through consistent cleaning, imputation, and exclusion rules, the possibility of information loss or imputation related uncertainty cannot be completely ruled out. These limitations should be kept in mind when interpreting the reported results. To evaluate predictive performance, the dataset was divided into training, validation, and test subsets, and cross validation was additionally used to assess robustness across different partitions of the data.

Model training was performed using the Adam optimizer with an initial learning rate of 0.001 and a batch size of 64. The learning rate was decayed during training to improve convergence stability, and model selection was based on validation performance. The proposed framework was trained in a multi stage manner, where the core predictive layers were first optimized to learn stable representations of the financial health relationship, followed by joint fine tuning of the full model. This strategy was adopted to balance predictive accuracy, numerical stability, and generalization under heterogeneous and partially noisy data conditions. Performance was evaluated using Accuracy, Recall, F1 score, and the Area Under the Receiver Operating Characteristic Curve (AUC), which together provide a balanced assessment of classification quality, sensitivity to positive cases, and ranking performance under class imbalance. To improve reliability, the reported results were obtained from repeated runs and cross validation summaries rather than from a single random split. This evaluation protocol allows the experimental results to reflect both predictive accuracy and robustness, which is particularly important in the present setting where financial instability and health system pressures may vary across contexts and time periods.

For transparency and reproducibility, additional implementation details are provided here. The dataset was partitioned into training, validation, and test subsets using a consistent split protocol, and repeated experiments were conducted under multiple random seeds to reduce sensitivity to initialization and data partitioning. Hyperparameters, including learning rate, batch size, and model specific regularization settings, were selected based on validation performance rather than test set optimization. Training was terminated according to validation based model selection and convergence stability criteria. In the present framework, the core predictive model consists of the probabilistic, temporal, and contextual modules described in Sections 3.1-3.3, whereas the scenario generation component is used as an auxiliary mechanism for robustness analysis and stress testing rather than as the main prediction backbone. The implementation was developed in PyTorch, and the manuscript now makes explicit the key settings necessary for replication, including optimization strategy, repeated runs, cross validation, and data preprocessing logic.

### 4.3 Comparison with SOTA methods

Before presenting the comparative results, it is useful to clarify how the empirical evaluation connects to the methodological framework introduced in Section 3. The comparison with baseline models is intended to assess whether the proposed unified framework provides superior predictive performance across the different financial and health related outcome domains considered in this study. The subsequent ablation analysis then examines whether the main methodological components introduced earlier namely the multidimensional probabilistic structure, temporal dynamics, and exogenous contextual adjustment make distinct contributions to that performance. In this way, the results section is organized to move from overall empirical validation of the framework to a more targeted assessment of how its core design elements support the study's predictive and analytical objectives.

To evaluate the effectiveness of the proposed framework, we compared it with a set of representative baseline methods commonly used in structured risk prediction and multivariate classification tasks. These baselines include conventional statistical learning models, tree based ensemble methods, and neural network based predictors, which together provide a more appropriate benchmark for the present problem than image oriented architectures. The comparison focuses on whether the proposed framework can better capture heterogeneous financial conditions, temporal variation, and contextual influences when predicting health economic and public health related outcomes.

Table 2 reports the comparative results on the Corporate Financial Risk Indicators Dataset and the Health Economics Expenditure Trends Dataset. Across all evaluation metrics, the proposed method achieves the best overall performance. On the corporate financial risk task, the model shows consistent gains in Accuracy, Recall, F1 score, and AUC, indicating that the integration of probabilistic inference, temporal dependence, and contextual variables improves the identification of risk sensitive patterns beyond what can be achieved by conventional baselines alone. On the health expenditure task, the performance advantage remains evident, suggesting that the proposed framework is also effective in modeling expenditure pressure and cost related outcomes under heterogeneous system conditions. These results support the view that the method is particularly well-suited to settings where financial stress and health system responses are jointly shaped by multiple interacting factors.

**Table 2:** Comparison of the proposed method with representative baseline models on the Corporate Financial Risk Indicators and Health Economics Expenditure Trends datasets.

Model	Corporate Financial Risk Indicators dataset				Health Economics Expenditure Trends dataset			
	Accuracy	Recall	F1 score	AUC	Accuracy	Recall	F1 score	AUC
Logistic regression (11)	85.67 ± 0.52	84.89 ± 0.63	84.12 ± 0.58	84.45 ± 0.47	88.45 ± 0.49	87.78 ± 0.61	87.03 ± 0.54	87.36 ± 0.50
Random forest (21)	86.92 ± 0.40	86.35 ± 0.51	85.62 ± 0.57	85.89 ± 0.42	89.54 ± 0.44	89.07 ± 0.56	88.29 ± 0.59	88.64 ± 0.46
XGBoost (22)	87.15 ± 0.48	86.58 ± 0.54	85.84 ± 0.60	86.12 ± 0.45	89.78 ± 0.47	89.21 ± 0.59	88.43 ± 0.62	88.78 ± 0.49
MLP (23)	86.45 ± 0.46	85.89 ± 0.52	85.16 ± 0.55	85.43 ± 0.44	89.12 ± 0.42	88.65 ± 0.54	87.87 ± 0.57	88.22 ± 0.45
LSTM (24)	86.78 ± 0.43	86.21 ± 0.50	85.48 ± 0.53	85.75 ± 0.41	89.36 ± 0.45	88.89 ± 0.57	88.11 ± 0.60	88.46 ± 0.48
GRU (25)	85.98 ± 0.50	85.42 ± 0.58	84.69 ± 0.56	84.96 ± 0.46	88.76 ± 0.48	88.19 ± 0.60	87.45 ± 0.58	87.78 ± 0.52
Ours	**89.82** **±0.37**	**89.25** **±0.45**	**88.52** **±0.42**	**88.79** **±0.40**	**91.54** **±0.38**	**91.01** **±0.49**	**90.33** **±0.46**	**90.68** **±0.39**

The bolded values represent the optimal values.

Table 3 further examines model performance on the Public Health Efficiency Metrics Dataset and the Financial Risk and Health Outcomes Correlation Dataset. The proposed framework again yields the strongest results across the reported metrics, demonstrating good generalizability across distinct but related outcome domains. In particular, the gains observed on the public health efficiency task suggest that the model is able to capture variation in service accessibility, delivery capacity, and efficiency related indicators, while the results on the broader financial risk and health related dataset indicate that the framework remains robust when the outcome space includes more diffuse socioeconomic and health relevant conditions.

**Table 3:** Comparison of the proposed method with representative baseline models on the Public Health Efficiency Metrics and Financial Risk and Health Outcomes Correlation datasets.

Model	Public Health Efficiency Metrics Dataset				Financial Risk and Health Outcomes Correlation Dataset			
	Accuracy	Recall	F1 score	AUC	Accuracy	Recall	F1 score	AUC
Logistic regression (11)	85.67 ± 0.52	84.89 ± 0.63	84.12 ± 0.58	84.45 ± 0.47	88.45 ± 0.49	87.78 ± 0.61	87.03 ± 0.54	87.36 ± 0.50
Random forest (21)	86.92 ± 0.40	86.35 ± 0.51	85.62 ± 0.57	85.89 ± 0.42	89.54 ± 0.44	89.08 ± 0.56	88.30 ± 0.59	88.65 ± 0.46
XGBoost (22)	87.15 ± 0.48	86.57 ± 0.54	85.84 ± 0.60	86.12 ± 0.45	89.78 ± 0.47	89.21 ± 0.59	88.43 ± 0.62	88.78 ± 0.49
MLP (23)	86.45 ± 0.46	85.89 ± 0.52	85.16 ± 0.55	85.43 ± 0.44	89.12 ± 0.45	88.66 ± 0.58	87.88 ± 0.57	88.23 ± 0.48
LSTM (23)	87.38 ± 0.43	86.81 ± 0.50	86.08 ± 0.53	86.35 ± 0.41	90.01 ± 0.42	89.54 ± 0.55	88.76 ± 0.56	89.11 ± 0.45
GRU (24)	86.78 ± 0.50	86.22 ± 0.57	85.49 ± 0.59	85.76 ± 0.46	89.34 ± 0.48	88.88 ± 0.60	88.10 ± 0.58	88.45 ± 0.51
Ours	**89.82** **±0.37**	**89.23** **±0.45**	**88.65** **±0.42**	**88.94** **±0.40**	**91.54** **±0.38**	**91.01** **±0.49**	**90.43** **±0.46**	**90.78** **±0.39**

The bolded values represent the optimal values.

These findings are closely aligned with the objectives of the present study. The consistent performance gains across the financial risk datasets support the predictive objective of improving the identification of heterogeneous and evolving corporate financial stress. The corresponding gains on health expenditure and public health efficiency datasets support the analytical objective of linking firm related financial conditions to multidimensional system level outcomes. The fact that the full framework performs better than simpler baselines and reduced specifications reinforces the decision support objective of the study, namely that modeling uncertainty, temporal evolution, and contextual dependence can provide more informative signals for early warning, resource prioritization, and resilience oriented health policy interpretation. A critical distinction should be made between the empirical findings of the present study and their broader interpretive implications. Empirically, the experiments support the conclusion that the proposed framework improves predictive performance and provides a more structured representation of the association between corporate financial conditions and multidimensional health related outcomes under the integrated dataset used in this study. By contrast, implications for health policy, public health planning, or system efficiency should be understood as cautious interpretive extensions of these findings rather than as directly validated policy effects. These broader implications are useful for decision support discussion, but they remain conditional on the limits of the data, the modeling assumptions, and the non- causal scope of the analysis.

### 4.4 Ablation study and robustness analysis

To examine the contribution of the major components of the proposed framework, we conducted an ablation study by removing one component at a time while keeping the remaining settings unchanged. The analysis focuses on three core elements of the model: the Multidimensional Probabilistic Framework, Temporal Dynamics Integration, and Exogenous Variable Integration. These components were selected because they correspond directly to the three substantive challenges highlighted in the methodological sections, namely heterogeneity in financial health relationships, temporal evolution of risk, and contextual dependence on macroeconomic and policy conditions.

Table 4 reports the ablation results for the Corporate Financial Risk Indicators dataset and the Health Economics Expenditure Trends dataset. The results show that removing the Multidimensional Probabilistic Framework leads to the largest decline in overall performance, indicating that explicit modeling of heterogeneous risk regimes and uncertainty is important for both financial risk identification and expenditure related outcome prediction. Removing the Temporal Dynamics Integration also reduces performance, particularly in Recall and F1 score, suggesting that time dependent variation in financial stress is relevant for capturing evolving expenditure pressure and related health system responses. The exclusion of Exogenous Variable Integration results in a smaller but still consistent performance loss, which indicates that contextual policy and macroeconomic conditions provide useful complementary information beyond firm level indicators alone.

**Table 4:** Ablation results of the proposed framework on the Corporate Financial Risk Indicators and Health Economics Expenditure Trends datasets.

Configuration	Corporate Financial Risk Indicators dataset				Health Economics Expenditure Trends dataset			
	Accuracy	Recall	F1 score	AUC	Accuracy	Recall	F1 score	AUC
w./o. Multidimensional probabilistic framework	87.45 ± 0.48	86.89 ± 0.55	86.12 ± 0.52	86.45 ± 0.44	90.12 ± 0.46	89.65 ± 0.58	88.87 ± 0.55	89.22 ± 0.47
w./o. Temporal dynamics integration	88.12 ± 0.42	87.56 ± 0.50	86.78 ± 0.49	87.11 ± 0.41	90.78 ± 0.43	90.31 ± 0.54	89.53 ± 0.52	89.88 ± 0.44
w./o. Exogenous variable integration	88.67 ± 0.40	88.09 ± 0.47	87.32 ± 0.46	87.65 ± 0.39	91.23 ± 0.41	90.76 ± 0.51	90.01 ± 0.49	90.36 ± 0.42
Ours	**89.82** **±0.37**	**89.25** **±0.45**	**88.52** **±0.42**	**88.79** **±0.40**	**91.54** **±0.38**	**91.01** **±0.49**	**90.33** **±0.46**	**90.68** **±0.39**

The bolded values represent the optimal values.

Table 5 presents the corresponding ablation results for the Public Health Efficiency Metrics Dataset and the Financial Risk and Health Outcomes Correlation Dataset. A similar pattern is observed across these tasks. The probabilistic component contributes most strongly to stable predictive performance, while temporal integration improves sensitivity to changing outcome conditions and the exogenous module enhances generalization across broader socioeconomic and health related contexts. Taken together, the ablation results support the interpretation that the proposed framework benefits from combining uncertainty modeling, dynamic structure, and contextual information. Rather than relying on a single modeling assumption, the framework performs best when these components are jointly incorporated, which is consistent with the multidimensional nature of the relationship between corporate financial risk and health system outcomes.

**Table 5:** Ablation results of the proposed framework on the Public Health Efficiency Metrics and Financial Risk and Health Outcomes Correlation datasets.

Configuration	Public Health Efficiency Metrics Dataset				Financial Risk and Health Outcomes Correlation Dataset			
	Accuracy	Recall	F1 score	AUC	Accuracy	Recall	F1 score	AUC
w./o. Multidimensional probabilistic framework	87.45 ± 0.48	86.89 ± 0.55	86.12 ± 0.57	86.39 ± 0.46	90.12 ± 0.44	89.65 ± 0.58	89.01 ± 0.60	89.34 ± 0.47
w./o. Temporal dynamics integration	88.23 ± 0.42	87.67 ± 0.50	86.90 ± 0.54	87.18 ± 0.43	90.78 ± 0.41	90.31 ± 0.53	89.67 ± 0.56	89.98 ± 0.44
w./o. Exogenous variable integration	88.56 ± 0.45	88.01 ± 0.52	87.24 ± 0.55	87.52 ± 0.44	91.02 ± 0.43	90.55 ± 0.56	89.91 ± 0.58	90.22 ± 0.46
Ours	**89.82** **±0.37**	**89.23** **±0.45**	**88.65** **±0.42**	**88.94** **±0.40**	**91.54** **±0.38**	**91.01** **±0.49**	**90.43** **±0.46**	**90.78** **±0.39**

The bolded values represent the optimal values.

Beyond component wise ablation, these reduced versions of the framework can also be interpreted as alternative model specifications for robustness assessment. In particular, the variants without temporal dynamics, without exogenous contextual adjustment, and without the multidimensional probabilistic component provide simpler specifications against which the full model can be compared. The fact that the proposed framework maintains consistent performance advantages across repeated runs, cross validation summaries, and these reduced specifications suggests that the main findings are not driven by a single modeling choice, but are robust to reasonable changes in model structure.

### 4.5 Model assumptions and limitations

The proposed framework relies on several assumptions that should be made explicit. We assume that the selected financial indicators provide an informative representation of latent corporate financial stress, even though no observed variable can fully capture all dimensions of firm vulnerability. We assume that the linkage between firm level financial risk and health related outcomes operates through intermediate organizational, labor market, insurance, and fiscal mechanisms, which are only partially observed in the available data. The dynamic component adopts a first order temporal specification as a tractable approximation of risk evolution, and the exogenous module assumes that macroeconomic and policy context can be partially represented through observed contextual variables. Fourth, the framework is intended to estimate structured predictive associations rather than to identify causal effects. These assumptions also imply several limitations. Because the empirical analysis integrates multiple public data sources collected at different levels and under different reporting standards, the resulting dataset may contain measurement error, temporal mismatch, and incomplete representation of intermediate transmission processes. The cross level linkage from firm conditions to system level health outcomes is modeled through harmonized associations rather than direct causal identification, and therefore the results should be interpreted with caution. The proposed model should thus be viewed as an analytical and decision support framework that captures risk sensitive patterns under uncertainty, rather than as a complete structural account of the underlying real world mechanisms.

## 5 Discussion

The findings of this study should be interpreted with appropriate caution. The empirical analysis is designed to estimate predictive and structural associations rather than to establish direct causal effects. The relationship between firm level financial risk and broader health system outcomes is understood to operate through intermediate institutional, socioeconomic, and policy related mechanisms, many of which cannot be fully identified within the present empirical design. Nevertheless, the framework remains relevant for public health planning and health economic analysis because it helps identify risk sensitive conditions that may warrant earlier monitoring, targeted resource allocation, and resilience oriented policy responses.

From a policy perspective, these findings have implications in at least two domains. For public health planning, the proposed framework can support earlier identification of risk sensitive conditions that may translate into expenditure pressure, service disruption, or reduced operational efficiency, thereby helping policymakers prioritize contingency resources, strengthen service continuity planning, and target vulnerable regions or sectors before pressures fully materialize. For financial regulation, the framework suggests that firm level financial instability may have broader system level relevance when it affects strategically connected employers, insurers, suppliers, or healthcare related organizations. This creates a rationale for integrating selected financial risk signals into sectoral monitoring, stress surveillance, and cross sector policy coordination, particularly in contexts where financial distress may propagate into public service or health system vulnerability. These implications should be understood as decision support considerations rather than direct regulatory prescriptions, but they illustrate how the analytical framework may inform both health sector resilience planning and broader financial oversight.

An important implication of the present framework is its potential relevance for low and middle income countries (LMICs), where health systems are often more vulnerable to financial shocks because of tighter fiscal space, weaker institutional buffers, and greater dependence on stable employment, insurance continuity, and external financing. In such settings, corporate financial distress may generate disproportionately large downstream effects on health expenditure pressure, service accessibility, continuity of provision, and the efficiency of resource use. The transmission logic developed in this study is therefore especially relevant to contexts in which limited public resources and fragmented service delivery structures amplify the system level consequences of economic instability. From this perspective, the proposed framework may be useful not only as a predictive tool, but also as a policy oriented early warning instrument for identifying vulnerable regions or sectors, prioritizing contingency resources, and strengthening resilience planning under resource constrained conditions. These implications should nevertheless be interpreted cautiously, and further validation with more granular longitudinal data from LMIC settings would be needed before drawing stronger policy conclusions.

In practical terms, the proposed predictive framework could be operationalized as a periodic monitoring and early warning tool for policymakers and health institutions. Updated financial, expenditure, and contextual indicators could be incorporated into a routine surveillance pipeline to generate risk scores, scenario based alerts, or ranked profiles of sectors and regions facing elevated vulnerability. Policymakers could use these outputs to inform contingency budgeting, targeted preparedness measures, and interagency coordination, while health institutions could use them to anticipate service disruption, adjust resource allocation, and strengthen continuity planning under financially adverse conditions. In this sense, the model is not intended to replace institutional judgment, but to provide a structured evidence layer that supports earlier and more coordinated decision making.

Several limitations should also be acknowledged. The current framework relies on integrated multi source data that may be affected by reporting heterogeneity, missing information, and incomplete measurement of intermediate transmission mechanisms. Future studies could strengthen the empirical design by incorporating more granular longitudinal data and by developing clearer identification strategies for separating predictive association from causal impact. Further extensions may also include richer temporal structures, alternative latent dependency specifications, and stronger mechanisms for scenario analysis and stress testing. In addition, future work could benefit from additional longitudinal, sector specific, or institution level sources that improve the measurement of intermediate transmission mechanisms and strengthen empirical alignment across firm level and health system variables. Finally, application and validation in more specific health system settings, such as hospital networks, insurance systems, regional public health infrastructures, or resource constrained environments, would help assess the practical relevance of the framework more directly.

## 6 Conclusions

This study examined how corporate financial risk may be associated with health economics and public health efficiency through a unified predictive and analytical framework. The central objective was not only to improve the estimation of financial risk, but also to clarify how firm level financial instability may translate into system level pressures on healthcare expenditure, service accessibility, continuity of provision, and the efficiency of resource utilization. To address this problem, the study developed an integrated framework combining the REHNM, the DRAM, and the SRHIA, thereby linking financial risk estimation, dynamic risk evolution, and policy oriented interpretation.

The findings indicate that the proposed framework provides consistent improvements in predictive performance across multiple datasets related to corporate financial conditions, health expenditure patterns, public health efficiency, and broader health related contextual outcomes. The comparative experiments and ablation analyses suggest that explicit modeling of heterogeneous risk regimes, incorporation of temporal dynamics, and adjustment for exogenous contextual conditions are particularly important for this problem setting. Taken together, these results support the usefulness of the proposed framework as a structured decision support tool for identifying conditions under which corporate financial instability may be associated with increased health system pressure and reduced public health efficiency.

## Data Availability

The original contributions presented in the study are included in the article/supplementary material, further inquiries can be directed to the corresponding author.
